# Perspectives on Rehabilitation Using Non-invasive Brain Stimulation Based on Second-Person Neuroscience of Teaching-Learning Interactions

**DOI:** 10.3389/fpsyg.2021.789637

**Published:** 2022-01-05

**Authors:** Naoyuki Takeuchi

**Affiliations:** Department of Physical Therapy, Akita University Graduate School of Health Sciences, Akita, Japan

**Keywords:** non-invasive brain stimulation, second-person neuroscience, instruction-based learning, metacognitive function, mentalizing, rehabilitation

## Abstract

Recent advances in second-person neuroscience have allowed the underlying neural mechanisms involved in teaching-learning interactions to be better understood. Teaching is not merely a one-way transfer of information from teacher to student; it is a complex interaction that requires metacognitive and mentalizing skills to understand others’ intentions and integrate information regarding oneself and others. Physiotherapy involving therapists instructing patients on how to improve their motor skills is a clinical field in which teaching-learning interactions play a central role. Accumulating evidence suggests that non-invasive brain stimulation (NIBS) modulates cognitive functions; however, NIBS approaches to teaching-learning interactions are yet to be utilized in rehabilitation. In this review, I evaluate the present research into NIBS and its role in enhancing metacognitive and mentalizing abilities; I then review hyperscanning studies of teaching-learning interactions and explore the potential clinical applications of NIBS in rehabilitation. Dual-brain stimulation using NIBS has been developed based on findings of brain-to-brain synchrony in hyperscanning studies, and it is delivered simultaneously to two individuals to increase inter-brain synchronized oscillations at the stimulated frequency. Artificial induction of brain-to-brain synchrony has the potential to promote instruction-based learning. The brain-to-brain interface, which induces inter-brain synchronization by adjusting the patient’s brain activity, using NIBS, to the therapist’s brain activity, could have a positive effect on both therapist-patient interactions and rehabilitation outcomes. NIBS based on second-person neuroscience has the potential to serve as a useful addition to the current neuroscientific methods used in complementary interventions for rehabilitation.

## Introduction

Teaching plays a central role in physiotherapy as physiotherapists instruct patients on how to improve their motor skills. Teaching-learning interactions are not a simple one-way transfer of information, but rather a complex process that involves speculation, evaluation, and mutual understanding between teachers and students (Rodriguez, 2013; Liu et al., 2019a). Speculating about the student’s behavior and learning state is a critical aspect of teaching and is related to the ability to understand the intentions of others, known as mentalizing (Strauss et al., 2014; Kline, 2015). In addition, teachers need metacognitive skills to monitor and regulate their own cognitive processes (Kline, 2015; Takeuchi et al., 2017). Similarly, students are not mere listeners or receivers of knowledge from their teachers. Students need metacognitive skills in order to learn, to monitor what they know, and understand what they are capable of doing (Chatzipanteli et al., 2015; Takeuchi et al., 2019). Students also need to consider the teacher’s intentions, even if they are not required to think deeply about the teacher’s mental state (Strauss et al., 2014; Zheng et al., 2018).

Many brain regions, including the prefrontal cortex (PFC), inferior frontal gyrus (IFG), temporo-parietal junction (TPJ), and medial precuneus are involved in metacognitive and mentalizing processes (Vogeley, 2017; Vaccaro and Fleming, 2018; Arioli and Canessa, 2019). These brain regions have been investigated as potential targets to modulate metacognitive and mentalizing skills *via* non-invasive brain stimulation (NIBS) techniques that can alter cortical excitability, such as repetitive transcranial magnetic stimulation (rTMS) and transcranial direct current stimulation (tDCS; Sowden et al., 2015; Chua et al., 2017; Gaynor and Chua, 2019; Martin et al., 2021). Recently, transcranial alternating current stimulation (tACS), which can non-invasively modulate oscillatory brain activity, has attracted attention as a promising technique for modulating social functions (Bramson et al., 2020). Metacognitive and mentalizing functions are impaired after brain damage, such as stroke and traumatic brain injury, and patients with brain damage often need rehabilitation (Martín-Rodríguez and León-Carrión, 2010; Al Banna et al., 2016; Nijsse et al., 2019; Yeo et al., 2021). Moreover, these social impairments are associated with poor rehabilitation outcomes. Therefore, NIBS approaches aimed at modulating metacognitive and mentalizing functions may enhance rehabilitation outcomes by facilitating therapist-patient interactions. However, to date, NIBS approaches used in rehabilitation have primarily focused on ameliorating patients’ motor and cognitive deficits (Takeuchi and Izumi, 2012; Ahorsu et al., 2021; Madrid and Benninger, 2021), and methods targeting interpersonal interactions in the teaching-learning process remain unexplored.

This review explores the potential for applying NIBS techniques in rehabilitation settings using second-person neuroscience approaches in teaching-learning interactions. First, I review studies using NIBS on single brains to enhance patients’ metacognitive, mentalizing, and imitation abilities. Next, I review brain-to-brain synchrony during teaching-learning interactions revealed by hyperscanning, which is a neuroimaging technique used to measure the activity of multiple brains simultaneously. Then, I discuss the potential for dual-brain stimulation using tACS to modulate interpersonal interactions related to instruction-based learning. Hyper-tACS in two individuals has been developed to induce inter-brain synchronized oscillation frequencies. The concurrent manipulation of brain activities helps elucidate the causal role of brain-to-brain synchrony in the teaching-learning process. Moreover, artificial induction of brain-to-brain synchrony may induce pro-social effects and enhance instruction-based learning. However, the use dual-brain stimulation to operate the therapist’s brain in real-word rehabilitation settings is unrealistic. Finally, to inform future physiotherapy research, considering therapist-patient interactions, I discuss the potential for using brain-to-brain interfaces, which allow two brains to mutually exchange decoded neural information, and the feedback of brain-to-brain synchrony to produce better rehabilitation outcomes.

## Single-Brain Approaches Using NIBS

Convergent evidence from a number of neuroimaging studies has suggested that the PFC plays a critical role in metacognitive processes (Fleming and Dolan, 2012; Vaccaro and Fleming, 2018). Hyperscanning studies of the teaching-learning process have shown that the PFC plays a central role in metacognitive function in both teachers and students (Takeuchi et al., 2017, 2019). Several studies have shown that NIBS over the PFC facilitates metacognitive processes, such as assessments of own knowledge. Chua et al. reported that excitatory anodal tDCS of the dorsolateral PFC improved the accuracy of participants’ general knowledge metamemory more than sham or active stimulation of the anterior temporal lobe (Chua et al., 2017). Gaynor et al. also reported that anodal tDCS of the anterior PFC improved the accuracy of participants’ judgments of learning in encoding low-frequency and inverted words, compared with sham or active stimulation of the dorsolateral PFC. However, anodal tDCS of the anterior PFC decreased participants’ judgments of learning accuracy in encoding high-frequency words relative to sham stimulation; these findings suggest that the role of the anterior PFC in the subjective level of confidence in metamemory varies according to task difficulty (Gaynor and Chua, 2019). Ryals et al. reported that bilateral continuous theta burst stimulation of the anterior PFC improved judgments of learning accuracy in an associative recognition task more than bilateral dorsolateral PFC or vertex stimulation (Ryals et al., 2016). Thus, NIBS of the PFC may be a potential adjuvant approach for facilitating instruction-based learning by enhancing metacognitive function; however, it should be noted that the effect of NIBS on the PFC varies greatly depending on the task and stimulation method.

The mentalizing system, which allows people to understand the intentions of others by making inferences about their thoughts and beliefs, has been reported to engage a range of brain regions, including the TPJ (especially the right hemisphere), the medial PFC (mPFC), and the medial precuneus (Vogeley, 2017; Arioli and Canessa, 2019). Sowden et al. showed that tDCS of the right TPJ (rTPJ) enhanced mentalizing performance. Excitatory anodal tDCS of the rTPJ improved participants’ accuracy at detecting whether a person was lying, specifically when the person’s own views conflicted with others’ views (Sowden et al., 2015). The mPFC is also a potential target that could be triggered to facilitate the mentalizing process using NIBS. Manfredi et al. reported that anodal tDCS of the mPFC improved participants’ abilities to recognize humorous situations that required mentalizing skills in order to be understood (Manfredi et al., 2020). Martin et al. investigated whether tDCS of the mPFC enhances mentalizing functions using the cross-cultural version of the Reading the Mind in the Eyes test. Anodal tDCS of the mPFC enhanced mentalizing performance, and this improvement was greater in participants with less cross-cultural contact (Martin et al., 2021).

In addition to the mentalizing system, the mirror neuron system that maps observed actions into the observer’s own motor representations is thought to be involved in the neural mechanism underlying understanding others’ intentions (Rizzolatti et al., 2014; Cole and Barraclough, 2018). Several brain imaging studies have reported that the IFG, premotor cortex, inferior parietal lobule (IPL), and superior parietal lobule are the main nodes of the mirror neuron system (Caspers et al., 2010; Molenberghs et al., 2012; Vogeley, 2017). Few studies have used NIBS to investigate the role of the mirror neuron system in the mentalizing process compared with the roles of the rTPJ and mPFC (Yao et al., 2021). However, several studies have reported that NIBS of the mirror neuron system could modulate imitation performance, which may be a promising technique to help patients acquire new motor skills during physiotherapy. Weiss et al. reported that anodal tDCS of the left IPL improved the matching task of hand gestures (Weiss et al., 2013). Vanbellingen et al. investigated whether dual-site stimulation, which consists of left IFG stimulation immediately followed by right IPL stimulation, modulated the imitation process. The results indicated that dual-site stimulation using continuous theta bursts enhanced the performance of gesture imitation compared with sham or left IFG stimulation (Vanbellingen et al., 2020). Consequently, the tACS approach to brain communication has attracted attention for modulating the imitation process. It has been reported that tACS of distant cortical regions can effectively modulate oscillatory phase synchrony and functional connectivity between the targeted brain regions *via* entrainment of brain oscillations (Cabral-Calderin and Wilke, 2020). Takeuchi et al. investigated whether the manipulation of brain communication in the frontoparietal mirror network altered imitation performance by applying gamma and theta phase-amplitude coupling (PAC) tACS to both the left IFG and left IPL. The application of in-phase theta-gamma PAC tACS over the left frontoparietal mirror network significantly improved participants’ gesture matching and meaningless gesture imitation performance relative to their baseline performance (Takeuchi et al., 2021). Theta-gamma PAC brain oscillations are hypothesized to coordinate activity in distant brain regions and induce effective communication during cognitive processing in humans (Canolty et al., 2006). Therefore, the increased synchrony between components of the left frontoparietal mirror network induced by in-phase theta-gamma PAC tACS may facilitate the imitation process by strengthening network efficiency. In addition to the mirror neuron system, it is thought that brain communication between distant regions, such as the mPFC and rTPJ, works together for the mentalizing process (Hill et al., 2017). It is worth considering whether dual-site stimulation to these distant brain regions enhances mentalizing ability more than single-site stimulation.

The results of NIBS studies suggest that manipulation of brain activities and communication related to metacognition, mentalizing, and imitation functions may be a potential approach to enhance the teaching-learning process in physiotherapy. However, the target of the NIBS approaches discussed in this chapter is mainly the single brain of a patient whose cognitive functions are impaired, although NIBS may also indirectly affect therapist-patient interactions. In the next chapter, I will focus on the NIBS approach used to target two brains in the context of therapist-patient interactions.

## Two-Brain Approaches Using NIBS

In the teaching-learning process, facial expressions and hand gestures, which are coupled to the teacher’s speech signals, may enhance the coupling of the student’s sensory system to the teacher’s motor system (Hasson et al., 2012; Hoehl et al., 2021). Social behaviors, such as mutual eye contact and joint attention during the learning process, also likely play a role in synchronizing neural responses in teachers and students (Dikker et al., 2014; Leong et al., 2017). Hyperscanning studies have demonstrated brain-to-brain synchrony during teaching-learning interactions (Zheng et al., 2018; Liu et al., 2019a; Pan et al., 2020). Using functional near-infrared spectroscopy, Liu et al. reported that the brain activities in the left PFC synchronized in teachers and students when they participated in an instruction-based learning task provided that students had prior knowledge. Moreover, brain-to-brain synchrony early in the teaching process is correlated with improved teaching effectiveness (Liu et al., 2019a). Pan et al. showed that brain-to-brain synchrony in the PFC and the superior temporal cortex during instruction-based learning tasks was correlated with better learning outcomes (Pan et al., 2020). Oscillatory synchronization through phase alignment may facilitate more efficient information transfer between brains, resulting in better teaching-learning interactions (Lakatos et al., 2019; Wass et al., 2020). Moreover, brain-to-brain synchrony may either cause or be caused by behavioral entrainment, cognitive alignment, and affective contagion during teaching-learning interactions, and these factors may work jointly to promote learning performance (Kingsbury and Hong, 2020; Pan et al., 2021a).

Artificial induction of brain-to-brain synchrony has the potential to promote instruction-based learning. It is well known that motor and sensory synchronization can cause brain-to-brain synchrony (Rennung and Göritz, 2016). Physical movement synchrony between teachers and students during instruction-based learning tasks has been associated with a higher perception of rapport and better learning performance in students (Zhou, 2012). However, brain-to-brain synchrony may be a byproduct of the behavioral and perceptual similarities between individuals during learning. Moreover, it should be noted that findings of brain-to-brain synchrony do not indicate a direct connection between brains. Therefore, to investigate the causal role of brain-to-brain synchrony in the teaching-learning process, it is essential to simultaneously manipulate brain activity in interacting partners and observe the subsequent effects on learning. At present, tACS is the main type of NIBS utilized for dual-brain stimulation to induce brain-to-brain synchrony in human studies (Novembre and Iannetti, 2021) because tACS has a feature that can modulate oscillatory phase synchrony and functional connectivity between targeted brain regions by entraining brain oscillations (Vosskuhl et al., 2018; Cabral-Calderin and Wilke, 2020). As an extension of the intra-brain to inter-brain communication approach, tACS applied simultaneously to two individuals (hyper-tACS) has been developed to modulate inter-brain synchronized oscillation frequency and artificially operate interpersonal interactions. Pan et al. investigated whether hyper-tACS of the IFG facilitated the teaching-learning process. They found that in-phase theta hyper-tACS in both the instructor and the learner augmented social interactive learning during a naturalistic song-learning task (Pan et al., 2021b). Moreover, hyper-tACS synchronized body movements between the instructor and the learner. Interpersonal movement synchrony is known to induce pro-social effects, such as rapport, cooperation, and affiliation (Keller et al., 2014; Reddish et al., 2016). These social factors might have a positive impact on the teaching-learning process and rehabilitation outcomes (Hall et al., 2010; Zee et al., 2013; Kinney et al., 2020). Considering these findings, hyper-tACS may enhance instruction-based learning in physiotherapy. In addition, the concurrent manipulation of brain activities using dual-brain stimulation methods, such as hyper-tACS, may help elucidate the causal role of therapist-patient brain-to-brain synchrony in the teaching-learning process, which cannot be achieved by hyperscanning and single-brain stimulation studies (Novembre and Iannetti, 2021).

However, there are several problems associated with hyper-tACS. First, tACS has been shown to be strongly affected by scalp sensory stimulation (Asamoah et al., 2019). Therefore, brain regions unrelated to cognitive functions must also be investigated as control sites in order to rule out the possibility that the observed positive effect of hyper-tACS on interpersonal interactions is merely derived from peripheral stimulation. Second, some doubt remains as to whether hyper-tACS can facilitate interpersonal interactions. Szymanski et al. showed that in-phase theta hyper-tACS of the left frontocentral and centroparietal sites during joint action impaired rather than improved performance during a dyadic drumming synchrony task (Szymanski et al., 2017). Although it is possible that the frequency and site of tACS were inappropriate for the dyadic task, this negative result suggests that the frequency entrained by tACS may differ between individuals, resulting in a reduction in dyadic synchrony. Third, participants’ brain activity during the teaching-learning process may not be completely synchronized in time and space. Zheng et al. reported that better learning performance was associated with stronger brain-to-brain synchrony between the rTPJ of the teacher and the anterior superior temporal cortex of the student when the teacher’s brain activity preceded that of the student (Zheng et al., 2018). Future studies aimed at promoting interpersonal interactions using hyper-tACS must take into account these temporal and spatial asymmetries in brain-to-brain synchrony.

Hyper-tACS may induce pro-social effects and enhance instruction-based learning *via* brain-to-brain synchrony. However, using NIBS to operate the therapist’s brain is not realistic in a real-world rehabilitation setting considering the ethical issues regarding manipulating healthy individuals’ brains. To further address the issues associated with hyper-tACS, I discuss in the final chapter of this paper the implications of using brain-to-brain interfaces, which allow the mutual exchange of decoded neural information between two brains, and the feedback of brain-to-brain synchrony to produce better rehabilitation outcomes.

## The Future of Physiotherapy Using Two-Person Neuroscience Approaches in Therapist-Patient Interactions

A new neural interface technology, i.e., the brain-to-brain interface, could induce brain-to-brain synchrony by operating the patient’s brain activity using NIBS and adjusting it to the therapist’s brain activity. A brain-to-brain interface allows two brains to mutually exchange decoded neural information with each other using a brain-computer interface that reads the sender’s brain activity and a computer-brain interface that transmits the neural information to the receiver’s brain using electrical stimulation (Nam et al., 2021). The direct transmission of information from a therapist’s to a patient’s brain *via* a brain-to-brain interface may facilitate instruction-based learning and more complex bidirectional therapist-patient interactions. In addition to brain stimulation, cross-brain neurofeedback is also a promising approach for manipulating brain-to-brain synchrony. A recent study showed that audiovisual feedback of brain-to-brain synchrony leads to increased inter-brain coupling (Dikker et al., 2021). Moreover, the study indicated that brain-to-brain synchrony was positively associated with social behaviors, such as eye contact and joint action. Considering these findings, NIBS assist and/or feedback of brain-to-brain synchrony may be a novel and useful rehabilitation technique to support patients’ learning by enhancing pro-social behavior. Feedback from brain-to-brain synchrony may also aid less experienced therapists (e.g., therapy students and novice therapists) in predicting the learning success of their patients. However, it is not clear whether the brains of therapists and patients synchronize during instruction-based learning in the same way that the brains of healthy subjects do. Future studies are required to determine whether brain-to-brain synchrony of therapists and patients alters according to patients’ learning performance.

Lastly, a cautionary point regarding therapeutic relationships must be considered before NIBS based on second-person neuroscience is used in physiotherapy. It is well known that the state of the therapist-patient relationship influences the effectiveness of psychotherapy (Koole and Tschacher, 2016). In physiotherapy, better therapeutic relationships are associated with improved rehabilitation outcomes (Hall et al., 2010; Kinney et al., 2020), and the therapeutic relationship between physiotherapists and patients is considered to be important in the field of physiotherapy (Søndenå et al., 2020; Bishop et al., 2021). Artificial induction of brain-to-brain synchrony using NIBS is expected to improve social interpersonal relationships in dyads of lovers (Liu et al., 2019b). However, the application of NIBS technologies to clinical fields to modulate the social interactions between therapists and patients raises important ethical issues and highlights the need for neuroethics guidelines (Cohen Kadosh et al., 2012; Ienca et al., 2017). Before NIBS approaches are used to improve the alliance between therapists and patients, it is necessary to first discuss the problems surrounding artificial manipulation of the therapist-patient relationship and ensure that the therapist’s neutrality toward the patient is maintained.

In conclusion, despite the translational potential of NIBS based on second-person neuroscience approaches to the rehabilitation field, the methodology remains in its infancy and poses several issues that will require greater innovation, both methodologically and ethically, before NIBS can be adopted into physiotherapy practices. This review identified the topics that will be instrumental in advancing neuroscientific knowledge of the instruction-based learning that is essential to physiotherapy.

## Author Contributions

The author confirms being the sole contributor of this work and has approved it for publication.

## Funding

This work was supported by a Grant-in-Aid for Scientific Research (no. 20K11278) from the Japan Society for the Promotion of Science.

## Conflict of Interest

The author declares that the research was conducted in the absence of any commercial or financial relationships that could be construed as a potential conflict of interest.

## Publisher’s Note

All claims expressed in this article are solely those of the authors and do not necessarily represent those of their affiliated organizations, or those of the publisher, the editors and the reviewers. Any product that may be evaluated in this article, or claim that may be made by its manufacturer, is not guaranteed or endorsed by the publisher.

## Abbreviations

IFG: inferior frontal gyrus
NIBS: non-invasive brain stimulation
IPL: inferior parietal lobule
PAC: phase-amplitude coupling
PFC: prefrontal cortex
rTMS: repetitive transcranial magnetic stimulation
tACS: transcranial alternating current stimulation
tDCS: transcranial direct current stimulation
TPJ: temporo-parietal junction
